# Large-scale assessment of socioeconomic, demographic and health system structures with US county excess mortality, 2020–2024

**DOI:** 10.64898/2026.07.04.26357291

**Published:** 2026-07-07

**Authors:** Michael Levitt, Ben Marten, Gal Oren, John P.A. Ioannidis

**Affiliations:** 1 Department of Structural Biology, Stanford University School of Medicine, Stanford, California, USA;; 2 Mortality.Watch, St. Petersburg, Florida, USA;; 3 Computer Science Department, Technion Israel Institute of Technology, Haifa, Israel;; 4 Meta-Research Innovation Center at Stanford (METRICS), Stanford University School of Medicine, Stanford, California, USA;; 5 Stanford Prevention Research Center, Department of Medicine, Stanford University School of Medicine, Stanford, California, USA;; 6 Department of Epidemiology and Population Health, Stanford University School of Medicine, Stanford, California, USA;; 7 Department of Biomedical Data Science, Stanford University School of Medicine, Stanford, California, USA

**Keywords:** COVID-19, excess mortality, US counties, social determinants, Area Health Resources File, dimensionality

## Abstract

Socioeconomic, demographic, and health system structures may have shaped COVID-19 pandemic impact across populations, but past analyses typically examined few factors. We systematically examined correlates of COVID-era excess mortality, considering 2,745 county-level variables of demography, race/ethnicity, income, insurance, education, employment, housing, and health system. Pearson correlation coefficients (CCs) were obtained for the most recent available pre-pandemic value against age-standardized county excess-death for each year during 2020–2024. Counties were population-weighted. Variables were grouped by meaning into 11 semantic super-clusters. Overall, 17.3% of variables reached at least a moderate correlation level (|CC| > 0.30) and 2.8% reached strong correlations (|CC| > 0.45). Strongest correlations were seen for college attainment (CC −0.54), uninsurance among adults 40–64 (+0.53), and high income (−0.53). At least moderate correlations were seen for 9.1% of variables in 2020 and 8.5% in 2021, but only 1.8%, 0%, and 1.3% in 2022, 2023, and 2024, respectively. Similar patterns of concentration of moderate correlations in the first two pandemic years appeared in both elderly and non-elderly populations. Of 472 variables with |CC| > 0.30, 362/395 moderate-band and 77/77 strong-band variables belonged to demography and socioeconomic super-clusters. Only 7% of health system variables reached |CC| > 0.30, versus 31% of socioeconomic and demographic variables. Using the most recent available value until 2023 or 2015, different population weighting, and Spearman correlations yielded similar results. Overall, these ecological analyses suggest strong relationships of socioeconomic structure and demographics rather than health-care resources/supply with excess mortality across US counties especially during 2020–2021.

## INTRODUCTION

The COVID-19 pandemic demonstrated large geographical variability in its footprint and revealed the high vulnerability of populations with disadvantaged status. County-level analyses of COVID-19 mortality have repeatedly implicated poverty, race, lack of insurance, and poor health-care access as correlates of adverse outcome (1–8), but the literature is fragmented. Each study has typically picked only a handful of predictors; moreover, conclusions can hinge on what analyses are performed and reported.

There is increasing interest in studying epidemiological exposures comprehensively at large scale, extending the omics paradigm that is dominant in genetics to diverse types of social, environmental, and other exposures (9–11). Moreover, increasing attention is given to robustness analyses, where large-scale assessments can be examined for their sensitivity to analytical choices (12, 13). Here, we combined these two principles to assess the footprint of diverse county-level features on COVID-19 excess deaths across US counties in 2020–2024.

Instead of pre-selecting predictors, we screened an exhaustive set of 2,745 county-level variables. Moreover, given that COVID-19 epidemiological estimates are known to be sensitive to the underlying data and modeling assumptions (14), we examined the robustness of the results considering different sets of variables collected until just prior to the pandemic (2019), during the pandemic, or 5 or more years before the pandemic; different population weighting; and different correlation metrics. Our analyses aimed to identify clusters and super-clusters of variables; examine how many variables have at least moderate correlations with excess deaths; and characterize whether some types of factors were more strongly related to excess deaths than others. Furthermore, we could assess whether the socioeconomic signature of excess deaths changed over time during the pandemic.

## METHODS

### County-level variables

County-level variables were drawn from the raw Area Health Resources File (AHRF) (15) (in contrast to pre-built composite area-level indices such as the Area Deprivation Index (16)), downloaded from the Health Resources and Services Administration data warehouse (https://data.hrsa.gov/data/download). The released file contains 7,236 county variable columns with different columns for the same variable measured at different years. Each variable carries an 8-character feature code (f + 7 digits) whose last two digits encode the data year (e.g. ‘10 = 2010, ‘18 = 2018, ‘22 = 2022). We applied a latest-year retention rule — for each base variable appearing in several years, we focused only on the most recent eligible year in the pre-pandemic “2019–2020” file, so as to consider the most recent pre-pandemic information, leaving 3,162 assembled columns. Of these, 2,745 were AHRF predictor variables that form the input to all downstream analyses (median year of reference 2018, only 40 variables from year 2020); the other 417 columns are not predictor variables — they were the mortality outcomes themselves (n=324), their population and death denominators (n=54), and geographic/administrative identifiers (n=39) (Supplementary Text; full list in excluded_columns_417.tsv). Figure S1 diagrams the full pipeline from source files to this variable set.

In two sensitivity analyses, we also considered the most recent year available for each variable (using the “2023–2024” file with median year 2022); and the most recent year up to 2015 (with median year 2015). Table S6 gives the per-data-year variable counts for the main and sensitivity analyses.

### Normalization (per-capita)

All variables were expressed per capita. Of the 2,745 predictors, 192 were already rates, percentages, or ratios (thus already per capita) and 2,526 were raw counts that were divided by the population of the respective year; the remaining 27 (county land area and a small number of administrative or metadata code variables) were left unscaled. The per-capita variables were then rescaled across counties to a 0–9,999 range (9,999 representing the per-capita maximum). All correlations below were computed on the normalized data.

### Mortality outcome

The outcome was the per-year, age-standardized excess-death percentage (asedx_p) for each pandemic year 2020–2024, asedx_p = (ased / ased_bl) − 1, where ased is the county’s age-standardized observed deaths in the year and ased_bl is its pre-pandemic age-standardized expected baseline derived from the average of the three pre-pandemic years 2017–2019. It is a proportion (e.g. 0.20 = 20% above baseline); counties with a zero baseline yield an undefined value and are set to missing. Values were taken from a purpose-built county-level mortality database derived from the US Centers for Disease Control and Prevention WONDER underlying-cause-of-death data (17); suppressed small county counts (1–9 deaths) were recovered by a difference method (national totals less the target county subtracted from overall national totals). Because the outcome was age-standardized, differences between counties in age structure are already removed from it. The main analysis used the all-age series; the same outcome was also computed for the under-65 (LT65) and 65-and-over (GE65) age groups, which we analyzed separately (Table S5).

### Association measure and effect-size bands

For each (variable, year) we computed a weighted Pearson correlation coefficient (CC) between the variable and the excess-death measure across counties, under population weighting (each county was weighted by its 2019 population). In sensitivity analyses, we also examined weighting by the 3/4 power and the square root of the population, as well as analyses using nonparametric Spearman correlation coefficients that do not have to assume linearity and can better accommodate extreme outliers.

We classified correlations based on their absolute value as weak (0–0.30), moderate (0.30–0.45), and strong (>0.45). We preferred focusing on the size of the correlation rather than the statistical significance, because given the large sample sizes involved p-values were expected to be highly significant even for what might be very weak correlations.

### Semantic clustering of variables

Variable names were embedded with a sentence-transformer neural language model (MPNet) (18, 19) and clustered using Ward’s agglomerative hierarchical clustering (Ward linkage) (20). A key step is name cleaning: before embedding we strip surface tokens that carry no subject information — age bands (_35_44), sex, patient-care/role suffixes, and Population_ / #_ / %_ / Hospital_with_ prefixes — so the embedding keyed on what is measured rather than incidental wording. For example, the six age-banded pulmonary-disease physician counts in the dataset (Pulmonary_Diseases_<35, _35_44, _45_54, _55_64, _65_74, _75+) and the eighteen psychiatry variants spanning age bands and practice roles (Psychiatry_Total, Psychiatry_Patient_Care_Office_Based, Psychiatry_Patient_Care_Hospital_Full_Time_Staff, Psychiatry_35_44, …) each collapse to a single embedded subject (“Pulmonary diseases”, “Psychiatry”); and a per-capita rate such as %_Males_40_64_without_Health_Insurance enters as “Males 40–64 without health insurance”. This process roughly doubled cluster cohesion as assessed by the silhouette coefficient — a cluster-cohesion score from −1 to 1, higher meaning tighter, better-separated clusters (21). We selected k = 120 clusters and then generated 11 super-clusters. The organizing unit for the presented analysis is the 11 super-clusters; the 120-cluster level is a finer index used for de-duplication and within-cluster ranking.

### Super-clusters

To generate the 11 super-clusters, the 120 cluster labels were themselves embedded, and a Ward linkage on the label cosine distance was cut at k = 11 to give contiguous super-clusters (renumbered top-to-bottom in dendrogram order). The 11 super-cluster names were written to match each branch’s membership.

### Reproducibility

The entire CC-dependent analysis layer regenerates from one script, code/run_standard_k120_w1.0.sh (steps A–K), writing all figures to figures_2745/ and tables to the project root and ward_sem_clean2_k120/. The k=120 clustering build, the 120 cluster labels, and the 11 super-cluster names are curated artifacts reused by the pipeline; everything downstream is scripted.

### Software

All analyses were implemented in Python. Scripts were developed iteratively with the assistance of a large language model (Claude, Anthropic) used as a coding aid: the investigators specified each analytical requirement, reviewed all generated code for correctness, and validated the outputs through multiple independent cross-checks, confirming that the |CC| moderate-association classification was stable across the 2019–2020, 2023–2024, and 2015 data vintages and that the age-standardized excess-death outcome reproduced the source CDC WONDER mortality totals. Variable-name embedding used the MPNet sentence-transformer; clustering used scikit-learn. The final scripts are deterministic and reproducible and are provided, together with the key data, in the public repository (see Data and code availability).

## RESULTS

### Structure of the variable space

**Figure 1.**
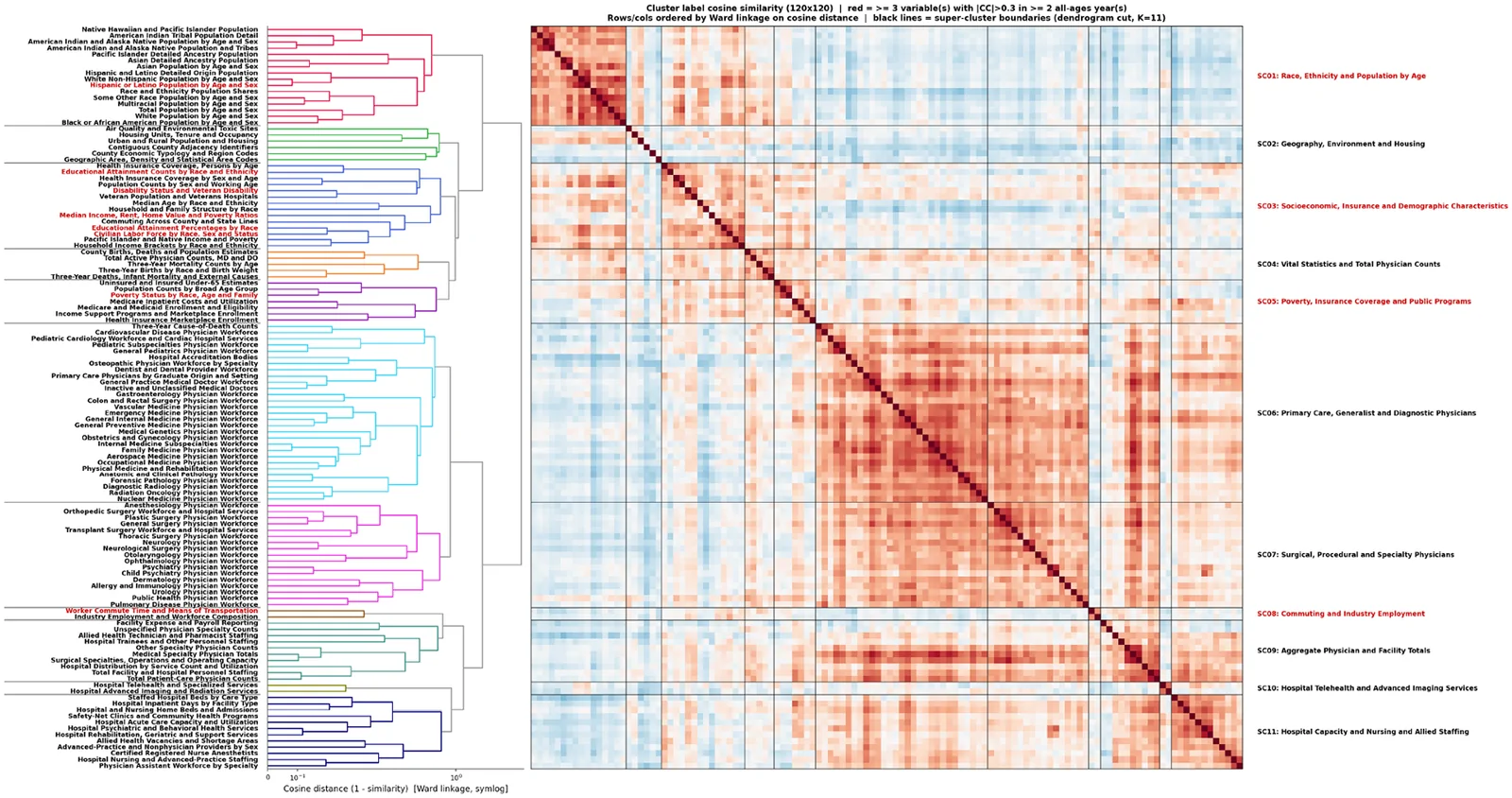
120×120 cluster-label cosine matrix with per-super-cluster-colored Ward dendrogram (symlog cosine-distance axis). Cluster labels are reddened when a cluster holds ≥3 variables each reaching the moderate band (|CC| > 0.30) in ≥2 years.

Cleaned-name clustering produced 120 cohesive semantic clusters (silhouette 0.42 at k=120; cluster sizes range 7–66 variables, median 18 — Figure S2). These clusters were aggregated into 11 super-clusters (Table S1; Figure 1); the full membership (each super-cluster and its constituent cluster names) is given in Table S3.

**Figure 2.**
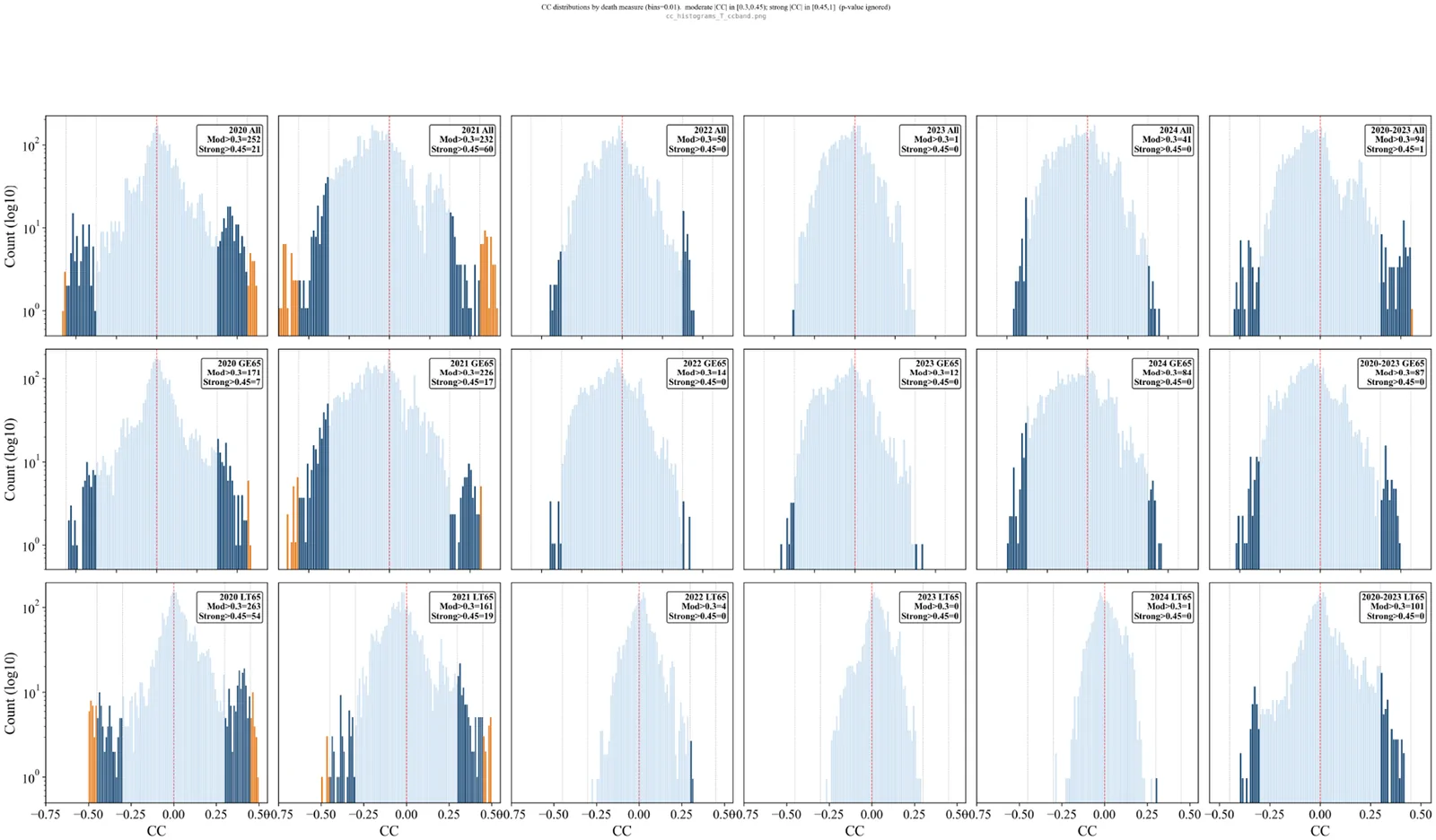
Per-variable correlation-coefficient (CC) distributions, 2019-vintage predictors, population-weighted. Columns: death years 2020–2024 plus pooled 2020–2023; rows: All / Ages ≥65 / Ages <65. log_10_ count on the y-axis; dark blue marks moderate (|CC| > 0.30), orange strong (|CC| > 0.45); red line at CC = 0.

Of the 2,745 screened variables, 2,727 carried a valid CC and a semantic-cluster assignment (the basis for Table 1); the remaining 18 were constant across all counties — all-zero counts of rare physician sub-specialties or services, or administrative name fields — giving zero variance and hence non-computable correlations. Across these 2,727 variables, the distribution of the maximum |CC| for any year in the period 2020–2024 had a median of 0.156, mean 0.181, 90th percentile 0.352, 95th percentile 0.406, and maximum 0.543. Overall, 17.3% of variables (472) reached the moderate correlation level (|CC| > 0.30) in at least one year and 2.8% (77) reached the strong correlation level (|CC| > 0.45) in at least one year. Figure 2 shows the full per-variable CC distributions by year and age group. As shown, moderate and strong correlations (|CC| > 0.30) appeared among variables in 2020 (9.1%) and 2021 (8.5%). In 2022 only some moderate correlations were seen (1.8%) and in 2023 essentially none (a single variable, 0.0%), while very few appeared in 2024 (1.3%). In the age strata, the patterns were very similar to the overall analysis, with moderate and strong correlations appearing in the first two years of the pandemic and then diminishing or even disappearing (even more prominently in the under-65 stratum) in subsequent years.

For interpretation we group the eleven super-clusters by subject matter into two families: socioeconomic/demographic (SC1, SC2, SC3, SC5, SC8) and health system (SC4, SC6, SC7, SC9, SC10, SC11). This grouping is by content, not a data-driven partition — a two-way cut of the cluster-label dendrogram would instead split the super-clusters into SC1–5 and SC6–11 (placing the physician-count super-cluster SC4 with the demographic block and employment SC8 with the workforce block).

### Socioeconomic and demographic factors

The variables with moderate or strong correlation were predominantly found in the demographic and socioeconomic family (Table 1). These five super-clusters — SC1 (Race, Ethnicity and Population by Age), SC2 (Geography, Environment and Housing), SC3 (Socioeconomic, Insurance and Demographic Characteristics), SC5 (Poverty, Insurance Coverage and Public Programs) and SC8 (Commuting and Industry Employment) — comprise 1,186 variables, 31% of which reach |CC| > 0.30; they account for 285 of the 395 variables in the moderate band and all 77 variables reaching the strong band.

The strongest correlations attained were with college attainment (CC −0.54 in 2021), uninsurance among adults 40–64 (+0.53 in 2021), and high income (−0.53 in 2021) in the overall analysis. In the ≥65 stratum the three strongest were all college-attainment measures (−0.50, −0.50 and −0.49, all in 2021); in the <65 stratum they were high household income (−0.50), the White non-Hispanic population aged 45–54 (−0.50), and college attainment (−0.50), with uninsurance among working-age adults just below (+0.50). The top six distinct moderately associated variables in each super-cluster (deduplicated to one per semantic cluster) are listed in Table S4, and the highest-|CC| variable in every one of the 120 clusters is given in the full per-cluster table (top_metric_per_cluster_2020_2024_w1.0.tsv, provided as Supplementary Data).

The pattern was internally consistent: affluence, education, and insurance were protective; poverty, uninsurance, disability, non-employment, and certain minority-population shares carried risk. Given that no single variable exceeded |CC| ≈ 0.54, no county characteristic explained more than ~29% of cross-county variance on its own (Figure S3).

### Health system factors

Across the 1,541 health system variables — the majority of the catalog (super-clusters 4, 6, 7, 9, 10, 11: physician counts, the physician workforce, and hospital capacity and facilities) — only 7.1% reached |CC| > 0.30 (with hospital-capacity and telehealth/imaging variables at 0–2%) versus 31% of the 1,186 socioeconomic and demographic variables (super-clusters 1, 2, 3, 5, 8) (Table 1). None of the health system variables reached a strong correlation. The six health system super-clusters contributed the bulk of the catalog (75 of 120 clusters) but little of the top signal. Physician-density variables correlated negatively with mortality but most of these correlations were weak. This is consistent with health system workforce supply tracking the same affluence/urbanicity gradient that drives the demographic signal, rather than an independent protective effect.

### Robustness in sensitivity analyses using different variable years

Variables were matched across vintages by base code (the f-code minus its last two-year digits): 2,396 base-matched between the main analysis and the older-data sensitivity analysis (latest year ≤ 2015) and 2,754 between the main analysis and the more-recent-data sensitivity analysis (the AHRF 2023–2024 release). Many matched variables were, however, identical by construction — their in-use vintage already qualified, so the backward cutoff or the forward value-swap left them unchanged — and such variables would be uninformative for a robustness test; we therefore restricted the comparison to the variables whose vintage value actually changed (1,328 for the ≤2015 comparison and 1,617 for the 2023–2024 comparison; the 1,068 and 1,137 identical variables are excluded). Among these genuinely refreshed variables the per-variable correlation with excess death was highly stable: each variable’s CC (2020–2024) in the main analysis tracked the older-data value at Spearman ρ = 0.95 (Pearson 0.94) and the more-recent-data value at ρ = 0.89 (Pearson 0.90); the moderate-association (|CC| > 0.30) classification agreed 98.0% and 95.1% (Table S2); and among the ~152 variables reaching |CC| > 0.30 in the main analysis, none reversed sign with the older data and only one did with the more recent data (Figure S6). Full distributions of correlations appear in Figure S4 and Figure S5.

### Sensitivity analysis on population weighting

To assess sensitivity to the weighting choice, we recomputed all correlations across a range of weight powers (each county weighted by its 2019 population raised to the power p; Table S7). The identity and ranking of the top correlates were unchanged throughout — uninsurance among adults 40–64 and college attainment remained the strongest — but the magnitudes scaled smoothly with p. At p = 0.75, a middle ground that roughly halves the dominance of the largest counties (Kish effective sample size (22) 712, versus 282 at full weighting), 10.6% of variables reached a max |CC| > 0.30 and 1.0% the strong band, versus 17.3% and 2.8% under the primary full-population weighting; the single strongest correlation was 0.49 versus 0.54. Square-root weighting (p = 0.5) brought the signal down to roughly the unweighted level (4.3% moderate, none strong). The headline effect sizes are therefore sensitive to how strongly large metropolitan counties are weighted, although the qualitative pattern — socioeconomic and demographic variables dominating, health system variables being weaker — holds at every weighting.

### Sensitivity analysis on the correlation method

The semantic super-cluster structure was unaffected by use of Spearman instead of Pearson correlation coefficients. The identity and ranking of the top correlates were also unchanged — uninsurance among adults 40–64 and college attainment remained the strongest. Spearman correlations tended to be somewhat larger than their Pearson counterparts: the single strongest correlation rose from |CC| = 0.54 to 0.65, the share of variables reaching a max |CC| > 0.30 rose from 17.3% to roughly 29%, and strong correlations rose from 2.8% to about 5.5% (Table S8). The qualitative pattern was preserved: socioeconomic and demographic variables again dominated, and the health system super-clusters remained far weaker — none reached the strong correlation level under Pearson, and under Spearman only three variables (one each in super-clusters 4, 6 and 11) just crossed it.

## DISCUSSION

We have evaluated at large scale the correlational footprint of 2,745 county-level variables against excess deaths during 2020–2024. Besides demographics, the social-determinant gradient that predicts many adverse health outcomes (with education (23), income (24), and insurance (25) being protective and poverty, uninsurance, disability, non-employment, and specific minority-population shares being risk-associated) offered the strongest signals for associations with excess deaths during the pandemic years. Health system supply and related variables (26) added a weak, mostly protective signal. Overall, even the strongest single variable topped out at |CC| ≈ 0.54; therefore, no county characteristic is close to deterministic, and the most honest summary may be the full-scale presentation of a multifactor socioeconomic gradient rather than any one specific driver.

A standing critique of ecological COVID studies is that associations are fragile to data choices. This problem largely jeopardizes studies that claim to identify highly specific predictors as being more important. However, in our analysis, the big picture where thousands of variables are considered remained robust to different analytical choices. Moreover, we demonstrated that results did not change substantially when we considered earlier or later measurements for the variables. This may reflect the fact that most of the county-level features do not change substantially over time. Disadvantaged counties remain disadvantaged and affluent ones remain affluent, with relatively few exceptions. Socioeconomic factors remain pervasive determinants of health and disease outcomes. We also found that the strength of the observed correlations largely tracked the magnitude of excess death itself. Excess death measures were strongest in 2020–21, decaying gradually with aggregate excess mortality returning to approximately zero by 2023–2024 (27). This socioeconomic disadvantage was most important in the early phase of the pandemic before vaccines were available and before the emergence of less lethal Omicron variants. The weaker correlations seen with health system factors may suggest that socioeconomic disadvantage may be more important than the exact capacity, resources, and specialization of the health system. People who are uninsured and lack access and those who suffer from marginalization may not be able to benefit much from improvements in the health system at the hospital level and workforce (28). Socioeconomic factors operate at a deeper root than can be fixed by traditional specialist hospital care and require special solutions and serious commitment that is usually lacking (29). We should acknowledge, however, that the health system variables that we used reflected the baseline structural endowment rather than real-time surge or ICU strain.

There is increasing interest in assessing the external exposome, the totality of exposures and potential risk factors to which people are exposed beyond their own genetics (30–33). Most applications examine traditional environmental exposures but may enrich the analysis with other determinants as well. These studies may vary in their selection of variables, use of individual versus group-level data or both, causality aspirations or lack thereof, and targeted outcomes. Regardless, they may offer some useful insights that extend beyond what analyses of single or few select determinants may provide. To our knowledge no previous study has examined such a large number of county-level variables in relationship to overall excess mortality. Given their complexity but also bird’s-eye view, ecological external exposome studies of this genre may prove particularly useful in offering high-level pictures about the potential architecture of risk of major outcomes (34). They may then be followed by targeted, more specific studies on factors or clusters of factors of particular interest and triangulation of evidence with additional methods (35,36). The clustering approach that we used offered some advantages in dealing with the very wide space of almost 3,000 variables. The super-clusters further allowed a more facile naming of the organized patterns of variables and high-level comparisons of different types of factors. However, it does have some limitations. A few variables may have been imperfectly placed, and the k=120 and k=11 choices were eventually analyst decisions, even though they were justified by silhouette metrics and dendrogram structure.

Several limitations should be discussed. The main limitation of this analysis is its ecological nature. Due to potential ecological fallacy (37), individual-level inferences should be avoided and causation claims with specific factors would be precarious. We argue, nevertheless, that once this is fully acknowledged, obtaining such a bird’s-eye view picture may be preferable to trying, in vain, to de-confound thousands of highly correlated and confounded factors. Socioeconomic risk factors travel together and effective interventions to fix them may affect many of them concurrently. We should acknowledge that reverse causality is also possible, especially in the sensitivity analysis that used county-level variables obtained during the pandemic years. Moreover, we did not use statistical significance testing as this would have yielded a huge number of nominally significant results of questionable meaning. The magnitude of the correlations is more meaningful. Their exact magnitude nevertheless depends on the nature of population weighting; and on whether linearity is assumed or not. Spearman correlations tended to be modestly stronger than Pearson correlations, which may reflect non-linear associations for some of the variables. We should also acknowledge that our approach would not necessarily detect associations that are U-shaped, inverse U-shaped or best-fit to other non-monotonic patterns (38). It is possible that some variables, especially health system-related ones, may exhibit such non-monotonic relationships. For example, poor individuals may suffer because of underuse of health care (39), while very wealthy individuals may overuse resources and thus have a risk of iatrogenic harms (40) and even addiction (41). However, for the vast number of informative relationships, monotonic assumptions are probably reasonable.

Acknowledging these caveats, we suggest that the study of socioeconomic and health system determinants of health would benefit from additional large-scale, bird’s-eye view analyses of comprehensive sets of many variables in diverse populations and settings. Comparison of the emerging patterns across different countries, time periods, and settings and with different robustness checks may highlight which factors are the most robust and persistent in relation to major outcomes of interest.

## Supplementary Material

1

## Figures and Tables

**Table 1.** Super-cluster association summary. For each of the 11 super-clusters, the count of variables whose maximum |CC| across the all-age years 2020–2024 (population-weighted, NORMED) reaches the moderate band (0.30 < |CC| < 0.45) and the strong band (|CC| > 0.45), with the percentage attaining |CC| > 0.30 and the mean of per-variable maximum |CC| (2,727 variables with a valid CC and a semantic-cluster assignment). Sorted by % |CC| > 0.30, descending.

Super Cluster ID	Super-cluster	Total variables	Moderate 0.30<|CC|<0.45	Strong |CC|>0.45	% |CC|>0.30	Mean |CC|	Strongest variable	CC (year)
3	Socioeconomic, Insurance and Demographic Characteristics	412	106	50	37.9	0.270	% adults 25+ with 4+ yr college, White non-Hisp.	−0.54 (2021)
5	Poverty, Insurance Coverage and Public Programs	175	42	18	34.3	0.232	% adults 40–64 without health insurance	+0.52 (2021)
8	Commuting and Industry Employment	48	10	5	31.2	0.224	% population did not work, 16–64	+0.50 (2021)
1	Race, Ethnicity and Population by Age	463	112	4	25.1	0.218	Hispanic/Latino (Ecuadorian) population	+0.50 (2020)
2	Geography, Environment and Housing	88	15	0	17.0	0.188	Owner-occupied housing units (count)	−0.43 (2020)
9	Aggregate Physician and Facility Totals	180	23	0	12.8	0.153	Medical-specialty physicians, 45–54	−0.37 (2021)
4	Vital Statistics and Total Physician Counts	88	11	0	12.5	0.147	Total M.D.s aged 55–64	−0.37 (2021)
6	Primary Care, Generalist and Diagnostic Physicians	590	52	0	8.8	0.155	Inactive female medical doctors	−0.42 (2021)
7	Surgical, Procedural and Specialty Physicians	266	18	0	6.8	0.169	Psychiatrists, 55–64	−0.33 (2021)
11	Hospital Capacity and Nursing and Allied Staffing	374	6	0	1.6	0.091	Education/health/social-assistance workers (count)	−0.36 (2021)
10	Hospital Telehealth and Advanced Imaging Services	43	0	0	0.0	0.077	Hispanic/Latino (all other), hospital pop.	+0.26 (2020)

## Data Availability

[https://github.com/michaellevitt/SCE-county-metrics]

## SIGNIFICANCE STATEMENT

COVID-19 mortality has been linked to poverty, race, and care access, and other diverse socioeconomic and population factors, but typically only a few factors have been assessed and reported each time. Screening the entire Area Health Resources File — 2,745 unselected county-level variables — we found excess death was ecologically associated with many variables (one out of six had absolute correlations > 0.30). Correlations reflected more strongly variables pertaining to demographics and socioeconomic structure rather than baseline hospital capacity or physician supply. The substantive correlation signals were seen almost exclusively in 2020 and 2021, but not in subsequent years. The patterns were robust in sensitivity analyses considering different years of measurement of the county-level variables, different population weighting, and different correlation metrics.
