# Supplementary material for: Large-scale assessment of socioeconomic, demographic and health system structures with US county excess mortality, 2020–2024
Source: medRxiv. 2026 Jul 7:2026.07.04.26357291. Preprint. [Version 1] doi: 10.64898/2026.07.04.26357291 (PMC13370557; doi:10.64898/2026.07.04.26357291)
Supplement: Supplementary file 1 [file NIHPP2026.07.04.26357291V1-supplement-1.pdf]

medRxiv preprint doi: https://doi.org/10.64898/2026.07.04.26357291; this version posted July 7, 2026. The copyright holder for this preprint (which was not certified by peer review) is the author/funder, who has granted medRxiv a license to display the preprint in perpetuity.
It is made available under a CC-BY 4.0 International license .

# SUPPLEMENT

## Supplementary Text

Significance mode. A p-value / log-probability (LP) mode exists in the pipeline but is dormant; the project default throughout this paper is the fixed |CC| effect-size rule (moderate |CC| > 0.30; strong |CC| > 0.45), a magnitude threshold rather than a statistical-significance test.

Excluded columns. Assembly retains 3,162 columns, of which 2,745 are AHRF predictor variables with a plain-English description — the input to all analysis. The remaining 417 columns are not predictor variables and were never candidates for the semantic or correlation analysis: 324 are the mortality / excess-death outcome series themselves (asedx_p and the analogous ASMR, CMR and life-expectancy measures, across years and age bands), 54 are the population and death denominators used to construct those outcomes, and 39 are geographic / administrative identifiers (FIPS and entity codes, CBSA and region codes, land area, and urban/rural population, housing and block tallies). No AHRF predictor variable was dropped for lacking a description. The full list of all 417 is provided as Supplementary Data (excluded_columns_417.tsv).

## Supplementary Data

Seven machine-readable files accompany the manuscript:

1. **Top_metric_per_cluster_2020_2024_w1.0.tsv** (the highest-|CC| variable in every one of the 120 clusters, 2020–2024);
2. **Table_S3_supercluster_cluster_names.tsv** (the full super-cluster → cluster membership listing, also rendered as Table S3 below);

medRxiv preprint doi: https://doi.org/10.64898/2026.07.04.26357291; this version posted July 7, 2026. The copyright holder for this preprint (which was not certified by peer review) is the author/funder, who has granted medRxiv a license to display the preprint in perpetuity.
It is made available under a CC-BY 4.0 International license .

3. **Table_S4_top6_metrics_per_supercluster.tsv** (the top six distinct moderately associated variables per super-cluster, rendered as Table S4);
4. **Table_S4A_collapsed_variants.tsv** (every near-duplicate variable removed in the Table S4 deduplication, listed under its representative);
5. **Table_S5_age_band_divergence.tsv** (moderate-association counts and max |CC| by year and age band, rendered as Table S5);
6. **Table_S6_vintage_composition.tsv** (variable counts by data-year for each AHRF release, rendered as Table S6); and
7. **Excluded_columns_417.tsv** (the 417 non-predictor columns excluded from analysis).

medRxiv preprint doi: https://doi.org/10.64898/2026.07.04.26357291; this version posted July 7, 2026. The copyright holder for this preprint (which was not certified by peer review) is the author/funder, who has granted medRxiv a license to display the preprint in perpetuity.
It is made available under a CC-BY 4.0 International license .

## Supplementary Tables

**Table S1.** The 11 super-clusters (Ward cut, k=11) and their size. Sizes count the 2,727 variables with a valid correlation (the 18 of the 2,745 screened predictors that lacked a usable cross-county correlation are omitted), matching the basis of Table 1.

| Super Cluster | Name | # clusters | # variables |
|---|---|---|---|
| 1 | Race, Ethnicity and Population by Age | 16 | 463 |
| 2 | Geography, Environment and Housing | 6 | 88 |
| 3 | Socioeconomic, Insurance and Demographic Characteristics | 14 | 412 |
| 4 | Vital Statistics and Total Physician Counts | 5 | 88 |
| 5 | Poverty, Insurance Coverage and Public Programs | 7 | 175 |
| 6 | Primary Care, Generalist and Diagnostic Physicians | 29 | 590 |
| 7 | Surgical, Procedural and Specialty Physicians | 17 | 266 |
| 8 | Commuting and Industry Employment | 2 | 48 |
| 9 | Aggregate Physician and Facility Totals | 10 | 180 |
| 10 | Hospital Telehealth and Advanced Imaging Services | 2 | 43 |
| 11 | Hospital Capacity and Nursing and Allied Staffing | 12 | 374 |

medRxiv preprint doi: https://doi.org/10.64898/2026.07.04.26357291; this version posted July 7, 2026. The copyright holder for this preprint (which was not certified by peer review) is the author/funder, who has granted medRxiv a license to display the preprint in perpetuity.
It is made available under a CC-BY 4.0 International license .

**Table S2.** Cross-vintage agreement on moderate-association status (|CC| > 0.30), restricted to the variables whose vintage value actually changed (1,068 backward and 1,137 forward base-matched variables that are identical by construction — not rolled back or not refreshed — are excluded as uninformative). The 2015 comparison is a retrospective cutoff of the 2019–2020 release, not an independent download; agreement is still partly carried by concordant sub-threshold variables.

| Comparison | Refreshed variables | Moderate in both | Neither in both | Agreement |
|---|---|---|---|---|
| 2015 vs 2019–2020 | 1,328 | 142 | 1,159 | 98.0% |
| 2019–2020 vs 2023–2024 | 1,617 | 132 | 1,405 | 95.1% |

medRxiv preprint doi: https://doi.org/10.64898/2026.07.04.26357291; this version posted July 7, 2026. The copyright holder for this preprint
(which was not certified by peer review) is the author/funder, who has granted medRxiv a license to display the preprint in perpetuity.
It is made available under a CC-BY 4.0 International license .

515 **Table S3.** The 11 super-clusters and their constituent 120 semantic clusters (cluster labels).

| Super Cluster | Name | Constituent clusters (semantic labels) |
|---|---|---|
| 1 | Race, Ethnicity and Population by Age | Asian Detailed Ancestry Population<br>Pacific Islander Detailed Ancestry Population<br>Race and Ethnicity Population Shares<br>Native Hawaiian and Pacific Islander Population<br>American Indian Tribal Population Detail<br>American Indian and Alaska Native Population by Age and Sex<br>American Indian and Alaska Native Population and Tribes<br>Total Population by Age and Sex<br>Asian Population by Age and Sex<br>White Population by Age and Sex<br>Some Other Race Population by Age and Sex<br>Multiracial Population by Age and Sex<br>Black or African American Population by Age and Sex<br>White Non-Hispanic Population by Age and Sex<br>Hispanic or Latino Population by Age and Sex<br>Hispanic and Latino Detailed Origin Population |
| 2 | Geography, Environment and Housing | Contiguous County Adjacency Identifiers<br>County Economic Typology and Region Codes<br>Geographic Area, Density and Statistical Area Codes<br>Air Quality and Environmental Toxic Sites<br>Housing Units, Tenure and Occupancy<br>Urban and Rural Population and Housing |
| 3 | Socioeconomic, Insurance and Demographic Characteristics | Health Insurance Coverage by Sex and Age<br>Health Insurance Coverage, Persons by Age<br>Population Counts by Sex and Working Age<br>Educational Attainment Counts by Race and Ethnicity<br>Educational Attainment Percentages by Race<br>Commuting Across County and State Lines<br>Civilian Labor Force by Race, Sex and Status<br>Pacific Islander and Native Income and Poverty<br>Household Income Brackets by Race and Ethnicity<br>Median Income, Rent, Home Value and Poverty Ratios<br>Median Age by Race and Ethnicity<br>Household and Family Structure by Race<br>Disability Status and Veteran Disability<br>Veteran Population and Veterans Hospitals |
| 4 | Vital Statistics and Total Physician Counts | Three-Year Mortality Counts by Age<br>Three-Year Births by Race and Birth Weight<br>Three-Year Deaths, Infant Mortality and External Causes<br>County Births, Deaths and Population Estimates<br>Total Active Physician Counts, MD and DO |
| 5 | Poverty, Insurance Coverage and Public Programs | Uninsured and Insured Under-65 Estimates<br>Population Counts by Broad Age Group<br>Poverty Status by Race, Age and Family |

medRxiv preprint doi: https://doi.org/10.64898/2026.07.04.26357291; this version posted July 7, 2026. The copyright holder for this preprint (which was not certified by peer review) is the author/funder, who has granted medRxiv a license to display the preprint in perpetuity.
It is made available under a CC-BY 4.0 International license .

| | | |
|---|---|---|
| | | Income Support Programs and Marketplace Enrollment<br>Health Insurance Marketplace Enrollment<br>Medicare Inpatient Costs and Utilization<br>Medicare and Medicaid Enrollment and Eligibility |
| 6 | Primary Care, Generalist and Diagnostic Physicians | Three-Year Cause-of-Death Counts<br>Dentist and Dental Provider Workforce<br>Hospital Accreditation Bodies<br>Emergency Medicine Physician Workforce<br>Osteopathic Physician Workforce by Specialty<br>General Internal Medicine Physician Workforce<br>General Preventive Medicine Physician Workforce<br>General Practice Medical Doctor Workforce<br>Inactive and Unclassified Medical Doctors<br>Internal Medicine Subspecialties Workforce<br>Family Medicine Physician Workforce<br>Primary Care Physicians by Graduate Origin and Setting<br>Obstetrics and Gynecology Physician Workforce<br>Pediatric Subspecialties Physician Workforce<br>General Pediatrics Physician Workforce<br>Pediatric Cardiology Workforce and Cardiac Hospital Services<br>Vascular Medicine Physician Workforce<br>Cardiovascular Disease Physician Workforce<br>Anatomic and Clinical Pathology Workforce<br>Forensic Pathology Physician Workforce<br>Diagnostic Radiology Physician Workforce<br>Aerospace Medicine Physician Workforce<br>Occupational Medicine Physician Workforce<br>Physical Medicine and Rehabilitation Workforce<br>Medical Genetics Physician Workforce<br>Radiation Oncology Physician Workforce<br>Nuclear Medicine Physician Workforce<br>Gastroenterology Physician Workforce<br>Colon and Rectal Surgery Physician Workforce |
| 7 | Surgical, Procedural and Specialty Physicians | Public Health Physician Workforce<br>Transplant Surgery Workforce and Hospital Services<br>Plastic Surgery Physician Workforce<br>Orthopedic Surgery Workforce and Hospital Services<br>General Surgery Physician Workforce<br>Thoracic Surgery Physician Workforce<br>Pulmonary Disease Physician Workforce<br>Psychiatry Physician Workforce<br>Child Psychiatry Physician Workforce<br>Otolaryngology Physician Workforce<br>Ophthalmology Physician Workforce<br>Anesthesiology Physician Workforce<br>Neurology Physician Workforce<br>Neurological Surgery Physician Workforce<br>Urology Physician Workforce<br>Dermatology Physician Workforce<br>Allergy and Immunology Physician Workforce |

medRxiv preprint doi: https://doi.org/10.64898/2026.07.04.26357291; this version posted July 7, 2026. The copyright holder for this preprint (which was not certified by peer review) is the author/funder, who has granted medRxiv a license to display the preprint in perpetuity.
It is made available under a CC-BY 4.0 International license .

| | | |
|---|---|---|
| 8 | Commuting and Industry Employment | Worker Commute Time and Means of Transportation<br>Industry Employment and Workforce Composition |
| 9 | Aggregate Physician and Facility Totals | Hospital Distribution by Service Count and Utilization<br>Facility Expense and Payroll Reporting<br>Unspecified Physician Specialty Counts<br>Allied Health Technician and Pharmacist Staffing<br>Hospital Trainees and Other Personnel Staffing<br>Total Facility and Hospital Personnel Staffing<br>Medical Specialty Physician Totals<br>Other Specialty Physician Counts<br>Surgical Specialties, Operations and Operating Capacity<br>Total Patient-Care Physician Counts |
| 10 | Hospital Telehealth and Advanced Imaging Services | Hospital Telehealth and Specialized Services<br>Hospital Advanced Imaging and Radiation Services |
| 11 | Hospital Capacity and Nursing and Allied Staffing | Certified Registered Nurse Anesthetists<br>Hospital Nursing and Advanced-Practice Staffing<br>Physician Assistant Workforce by Specialty<br>Allied Health Vacancies and Shortage Areas<br>Hospital Inpatient Days by Facility Type<br>Staffed Hospital Beds by Care Type<br>Hospital and Nursing-Home Beds and Admissions<br>Advanced-Practice and Nonphysician Providers by Sex<br>Hospital Psychiatric and Behavioral Health Services<br>Safety-Net Clinics and Community Health Programs<br>Hospital Rehabilitation, Geriatric and Support Services<br>Hospital Acute Care Capacity and Utilization |

medRxiv preprint doi: https://doi.org/10.64898/2026.07.04.26357291; this version posted July 7, 2026. The copyright holder for this preprint (which was not certified by peer review) is the author/funder, who has granted medRxiv a license to display the preprint in perpetuity.
It is made available under a CC-BY 4.0 International license .

**Table S4.** Top six moderately associated (|CC| > 0.30) distinct variables per super-cluster, with the signed CC at the year of maximum |CC| (Yr). Deduplicated: within each super-cluster only one representative — the highest-|CC| member — is shown per k=120 semantic cluster, so the listed variables are distinct measures rather than age/sex/race/vintage variants of the same one. The Collapsed column gives the number of other moderately associated variables in that cluster that the representative stands for; those variants are enumerated in Table_S4A_collapsed_variants.tsv (Supplementary Data). Where a super-cluster has fewer than six such clusters, fewer than six rows appear. Same basis and super-cluster ordering as Table 1; SC10 (Hospital Telehealth and Advanced Imaging Services) has no variable reaching |CC| > 0.30. Variable names are AHRF descriptions (verbatim, including source spellings).

| SC | Super-cluster (# variables) | # | Representative variable | CC | Yr | Collapsed |
|---|---|---|---|---|---|---|
| 3 | Socioeconomic, Insurance and Demographic Characteristics (412) | 1 | Per 25+ with 4+ Year College White non Hispanic | -0.54 | 2021 | 18 |
| | | 2 | % Female 40 64 without Health Insurance | +0.53 | 2021 | 11 |
| | | 3 | #White non Hispanic Household Income $100 000+ | -0.53 | 2021 | 19 |
| | | 4 | Personal Persons 25+ with 4+ Years College White | -0.52 | 2021 | 11 |
| | | 5 | Ratio of Income to Poverty Level 2.0+ | -0.51 | 2021 | 12 |
| | | 6 | Employed Female in Civilian Labor Force | -0.51 | 2021 | 31 |
| 5 | Poverty, Insurance Coverage and Public Programs (175) | 1 | % 40 64 without Health Insurance | +0.52 | 2021 | 21 |
| | | 2 | Related Child Below Poverty Level | +0.50 | 2021 | 22 |
| | | 3 | Inpatient Per Capita Actual Medicare Cost | +0.50 | 2020 | 2 |
| | | 4 | Medicare Fee for Service Beneficiaries Average Hierarchical Condition Categories Score | +0.48 | 2020 | 2 |
| | | 5 | Persons <65 < | +0.33 | 2022 | 8 |

medRxiv preprint doi: https://doi.org/10.64898/2026.07.04.26357291; this version posted July 7, 2026. The copyright holder for this preprint (which was not certified by peer review) is the author/funder, who has granted medRxiv a license to display the preprint in perpetuity.
It is made available under a CC-BY 4.0 International license .

| | | | | | | |
|---|---|---|---|---|---|---|
| 8 | Commuting and Industry Employment (48) | 1 | % Population Did Not Work 16 64 | +0.50 | 2021 | 5 |
| | | 2 | # Workers 16 and Over | -0.48 | 2021 | 8 |
| 1 | Race, Ethnicity and Population by Age (463) | 1 | Hispanic Latino Origin Ecuadorian Population | +0.50 | 2020 | 16 |
| | | 2 | Population Black African Hispanic Latino Female | +0.48 | 2020 | 1 |
| | | 3 | American Indian South American Population | +0.45 | 2020 | 2 |
| | | 4 | Population Some Other Race Female 65 74 | +0.45 | 2020 | 19 |
| | | 5 | Population White Non Hispanic Female 55 59 | -0.42 | 2020 | 25 |
| | | 6 | Asian Bangladeshi Population | +0.42 | 2020 | 2 |
| 2 | Geography, Environment and Housing (88) | 1 | # Owner Occupied Housing Units | -0.43 | 2020 | 3 |
| | | 2 | Population Density per Square Mile | +0.42 | 2020 | 1 |
| | | 3 | Persistent Poverty Typology Code | +0.38 | 2020 | 6 |
| | | 4 | Census Urbanized Areas Housing | -0.31 | 2024 | 1 |
| 9 | Aggregate Physician and Facility Totals (180) | 1 | Medical Specialty Total 45 54 | -0.37 | 2021 | 9 |
| | | 2 | M.D.'s Total Other | -0.36 | 2021 | 2 |
| | | 3 | Other Specialty Total 45 54 | -0.35 | 2021 | 7 |
| | | 4 | Surgical Specialty Total 45 54 | -0.33 | 2021 | 1 |
| 4 | Vital Statistics and Total Physician Counts (88) | 1 | Total M.D.'s 55 64 | -0.37 | 2021 | 9 |
| | | 2 | Unemployment Rate 16+ | +0.34 | 2021 | 0 |
| 6 | Primary Care, Generalist and Diagnostic Physicians (590) | 1 | Medical Doctors Inactive Female | -0.42 | 2021 | 5 |
| | | 2 | Physician Primary Care Patient Care | -0.41 | 2021 | 10 |
| | | 3 | Dentists Total Private Practice | -0.39 | 2021 | 9 |
| | | 4 | General Internal Medical Medicine 45 54 | -0.38 | 2021 | 7 |
| | | 5 | Pediatrics General 45 54 | -0.34 | 2021 | 4 |
| | | 6 | Emergency Medical Medicine Patient Care Hospital Full Time Staff | -0.34 | 2021 | 3 |

medRxiv preprint doi: https://doi.org/10.64898/2026.07.04.26357291; this version posted July 7, 2026. The copyright holder for this preprint (which was not certified by peer review) is the author/funder, who has granted medRxiv a license to display the preprint in perpetuity.
It is made available under a CC-BY 4.0 International license .

| | | | | | | |
|---|---|---|---|---|---|---|
| 7 | Surgical, Procedural and Specialty Physicians (266) | 1 | Psychiatry 55 64 | -0.33 | 2021 | 2 |
| | | 2 | Ophthalmology Patient Care Office Based | -0.32 | 2021 | 2 |
| | | 3 | Child Psychiatric Patient Care Office Based | -0.32 | 2021 | 3 |
| | | 4 | Dermatology Patient Care Office Based | -0.32 | 2021 | 2 |
| | | 5 | Allergy & Immunology Total | -0.31 | 2021 | 2 |
| | | 6 | Anesthesiology 45 54 | -0.31 | 2021 | 1 |
| 11 | Hospital Capacity and Nursing and Allied Staffing (374) | 1 | # Education Healthcare Social Assist Work | -0.36 | 2021 | 0 |
| | | 2 | Dentists with National Provider Identifier | -0.35 | 2021 | 4 |
| 10 | Hospital Telehealth and Advanced Imaging Services (43) | - | (none reaching the moderate band) | - | - | - |

medRxiv preprint doi: https://doi.org/10.64898/2026.07.04.26357291; this version posted July 7, 2026. The copyright holder for this preprint (which was not certified by peer review) is the author/funder, who has granted medRxiv a license to display the preprint in perpetuity.
It is made available under a CC-BY 4.0 International license .

**Table S5.** Age-band divergence over the pandemic. Counts of moderately associated variables (|CC| > 0.30, strict) and the single strongest |CC| for the all-age, under-65 (LT65) and 65-and-over (GE65) excess-death outcomes, by year (population-weighted, 2019-vintage predictors). The under-65 signal is confined to 2020–2021; the ≥65 signal persists and re-strengthens through 2024. (A displayed max of 0.30 for all-age 2023 is rounding — no variable strictly exceeds 0.30, hence zero in that band.)

| Year | # sig (all) | # sig (<65) | # sig (≥65) | max\|CC\| (all) | max\|CC\| (<65) | max\|CC\| (≥65) |
|---|---|---|---|---|---|---|
| 2020 | 248 | 259 | 167 | 0.50 | 0.50 | 0.46 |
| 2021 | 232 | 161 | 226 | 0.54 | 0.50 | 0.50 |
| 2022 | 50 | 4 | 14 | 0.36 | 0.32 | 0.35 |
| 2023 | 1 | 0 | 10 | 0.30 | 0.28 | 0.36 |
| 2024 | 35 | 1 | 78 | 0.36 | 0.31 | 0.39 |

medRxiv preprint doi: https://doi.org/10.64898/2026.07.04.26357291; this version posted July 7, 2026. The copyright holder for this preprint (which was not certified by peer review) is the author/funder, who has granted medRxiv a license to display the preprint in perpetuity.
It is made available under a CC-BY 4.0 International license .

**Table S6.** Data-year composition of the variables actually used in each analysis, after the latest-year retention rule (one most-recent year retained per variable). Columns: the ≤2015 backward-cutoff vintage, the 2019–2020 main analysis, and the 2023–2024 forward vintage; each row gives the number of retained variables carrying that data year (f-coded predictors plus the non-f-code count, rate and land-area predictors; pure geographic/administrative identifier codes are excluded). The 2019–2020 column is dominated by 2018, and secondarily 2010 and 2014 years; the 2023–2024 column is dominated by year 2022. Totals 2,395 / 2,754 / 2,754. The 2019–2020 file includes only ≤2019 variables, except for 40 variables from 2020: 29 are 2020 decennial-Census predictors — county population, housing-unit and land-area counts together with their densities and urban/rural shares, for which the 2020 Census is the most recent available measurement — and 11 are administrative geographic-identifier codes and names (Core-Based and Combined Statistical Area and Metropolitan Division labels), several of the latter being among the zero-variance variables dropped from the analysis.

| Data year | ≤2015 vintage | 2019–2020 vintage (main analysis) | 2023–2024 vintage |
|---|---|---|---|
| 1960 | 1 | 1 | 1 |
| 1970 | 1 | 1 | 1 |
| 1976 | 1 | 1 | 1 |
| 1990 | 1 | 1 | 1 |
| 2000 | 7 | 7 | 7 |
| 2001 | 2 | 2 | 2 |
| 2003 | 13 | 13 | 13 |
| 2004 | 4 | 4 | 4 |
| 2010 | 589 | 589 | 589 |
| 2012 | 11 | 11 | 11 |
| 2013 | 19 | 19 | 18 |
| 2014 | 345 | 345 | 345 |
| 2015 | 1361 | 12 | 12 |
| 2016 | 0 | 71 | 68 |

medRxiv preprint doi: https://doi.org/10.64898/2026.07.04.26357291; this version posted July 7, 2026. The copyright holder for this preprint (which was not certified by peer review) is the author/funder, who has granted medRxiv a license to display the preprint in perpetuity.
It is made available under a CC-BY 4.0 International license .

| | | | |
|---|---|---|---|
| 2017 | 0 | 3 | 1 |
| 2018 | 0 | 1539 | 6 |
| 2019 | 0 | 95 | 2 |
| 2020 | 40 | 40 | 32 |
| 2021 | 0 | 0 | 78 |
| 2022 | 0 | 0 | 1456 |
| 2023 | 0 | 0 | 87 |
| 2024 | 0 | 0 | 19 |
| Total | 2395 | 2754 | 2754 |

medRxiv preprint doi: https://doi.org/10.64898/2026.07.04.26357291; this version posted July 7, 2026. The copyright holder for this preprint (which was not certified by peer review) is the author/funder, who has granted medRxiv a license to display the preprint in perpetuity.
It is made available under a CC-BY 4.0 International license .

**Table S7.** Sensitivity of the variable–death correlations to the county weighting scheme. Each county is weighted by its 2019 population raised to the power p (p = 0 unweighted; p = 1.0 the primary full-population analysis). For each scheme: the Kish effective sample size; the percentage of the 2,727 analyzed variables whose maximum |CC| over 2020–2024 reaches the moderate (> 0.30) and strong (> 0.45) bands; the median of that maximum |CC|; and the single largest |CC|. The identity and ranking of the top correlates (uninsurance among adults 40–64; college attainment) are unchanged across schemes — only the magnitudes scale with p. The unweighted row is sensitive to the inclusion of very small, noisy counties (applying a legacy minimum-population floor raises the unweighted moderate fraction to ~3.6%).

| Weight power p | County weighting | N_eff | % \|CC\|>0.30 | % \|CC\|>0.45 | Median max \|CC\| | Max \|CC\| |
|---|---|---|---|---|---|---|
| 0 | Unweighted | 3,139 | 0.3% | 0% | 0.066 | 0.370 |
| 0.5 | Square-root of population | 1,595 | 4.3% | 0% | 0.107 | 0.440 |
| 0.75 | Population^0.75 | 712 | 10.6% | 1.0% | 0.131 | 0.491 |
| 1.0 | Full population (primary) | 282 | 17.3% | 2.8% | 0.156 | 0.543 |

medRxiv preprint doi: https://doi.org/10.64898/2026.07.04.26357291; this version posted July 7, 2026. The copyright holder for this preprint (which was not certified by peer review) is the author/funder, who has granted medRxiv a license to display the preprint in perpetuity.
It is made available under a CC-BY 4.0 International license .

**Table S8.** Sensitivity of the variable–death correlations to the choice of correlation coefficient, by super-cluster. For each of the 11 semantic super-clusters: the number of variables (N) and, under population-weighted Pearson (P) and population-weighted Spearman (S) correlation, the percentage of variables whose maximum |CC| over the all-age years 2020–2024 reaches the moderate band (> 0.30), the number reaching the strong band (> 0.45), and the single largest |CC| (2,727 variables with a valid CC, as in Table 1). Spearman values are generally somewhat larger, but the qualitative pattern is preserved: the socioeconomic and demographic super-clusters dominate, accounting for 145 of the 148 strong-band variables under Spearman; among the health system super-clusters none contains a strong-band variable under Pearson and only three do under Spearman (a single variable each in super-clusters 4, 6 and 11).

| Super-cluster | N | % mod (P) | % mod (S) | strong n (P) | strong n (S) | max \|CC\| (P) | max \|CC\| (S) |
|---|---|---|---|---|---|---|---|
| 1. Race, Ethnicity and Population by Age | 463 | 25.1 | 35.2 | 4 | 4 | 0.50 | 0.46 |
| 2. Geography, Environment and Housing | 88 | 17.0 | 35.2 | 0 | 0 | 0.43 | 0.44 |
| 3. Socioeconomic, Insurance and Demographic Characteristics | 412 | 37.9 | 54.4 | 50 | 90 | 0.54 | 0.63 |
| 4. Vital Statistics and Total Physician Counts | 88 | 12.5 | 37.5 | 0 | 1 | 0.37 | 0.49 |
| 5. Poverty, Insurance Coverage and Public Programs | 175 | 34.3 | 49.1 | 18 | 47 | 0.52 | 0.65 |
| 6. Primary Care, Generalist and Diagnostic Physicians | 590 | 8.8 | 18.3 | 0 | 1 | 0.42 | 0.48 |
| 7. Surgical, Procedural and Specialty Physicians | 266 | 6.8 | 14.7 | 0 | 0 | 0.33 | 0.39 |
| 8. Commuting and Industry Employment | 48 | 31.2 | 39.6 | 5 | 4 | 0.50 | 0.55 |
| 9. Aggregate Physician and Facility Totals | 180 | 12.8 | 20.6 | 0 | 0 | 0.37 | 0.42 |
| 10. Hospital Telehealth and Advanced Imaging Services | 43 | 0.0 | 4.7 | 0 | 0 | 0.26 | 0.34 |

medRxiv preprint doi: https://doi.org/10.64898/2026.07.04.26357291; this version posted July 7, 2026. The copyright holder for this preprint (which was not certified by peer review) is the author/funder, who has granted medRxiv a license to display the preprint in perpetuity.
It is made available under a CC-BY 4.0 International license .

| | | | | | | | |
|---|---|---|---|---|---|---|---|
| 11. Hospital Capacity and Nursing and Allied Staffing | 374 | 1.6 | 5.1 | 0 | 1 | 0.36 | 0.45 |

medRxiv preprint doi: https://doi.org/10.64898/2026.07.04.26357291; this version posted July 7, 2026. The copyright holder for this preprint (which was not certified by peer review) is the author/funder, who has granted medRxiv a license to display the preprint in perpetuity.
It is made available under a CC-BY 4.0 International license .

## Supplementary Figures

**Figure S1.** *Pipeline overview, from source files (AHRF technical documentation, AHRF data, CDC WONDER mortality) to the analysis variable set and master data table. Documents the reduction from 7,236 raw AHRF variable columns (all vintages) to the 2,745 described variables used downstream.*

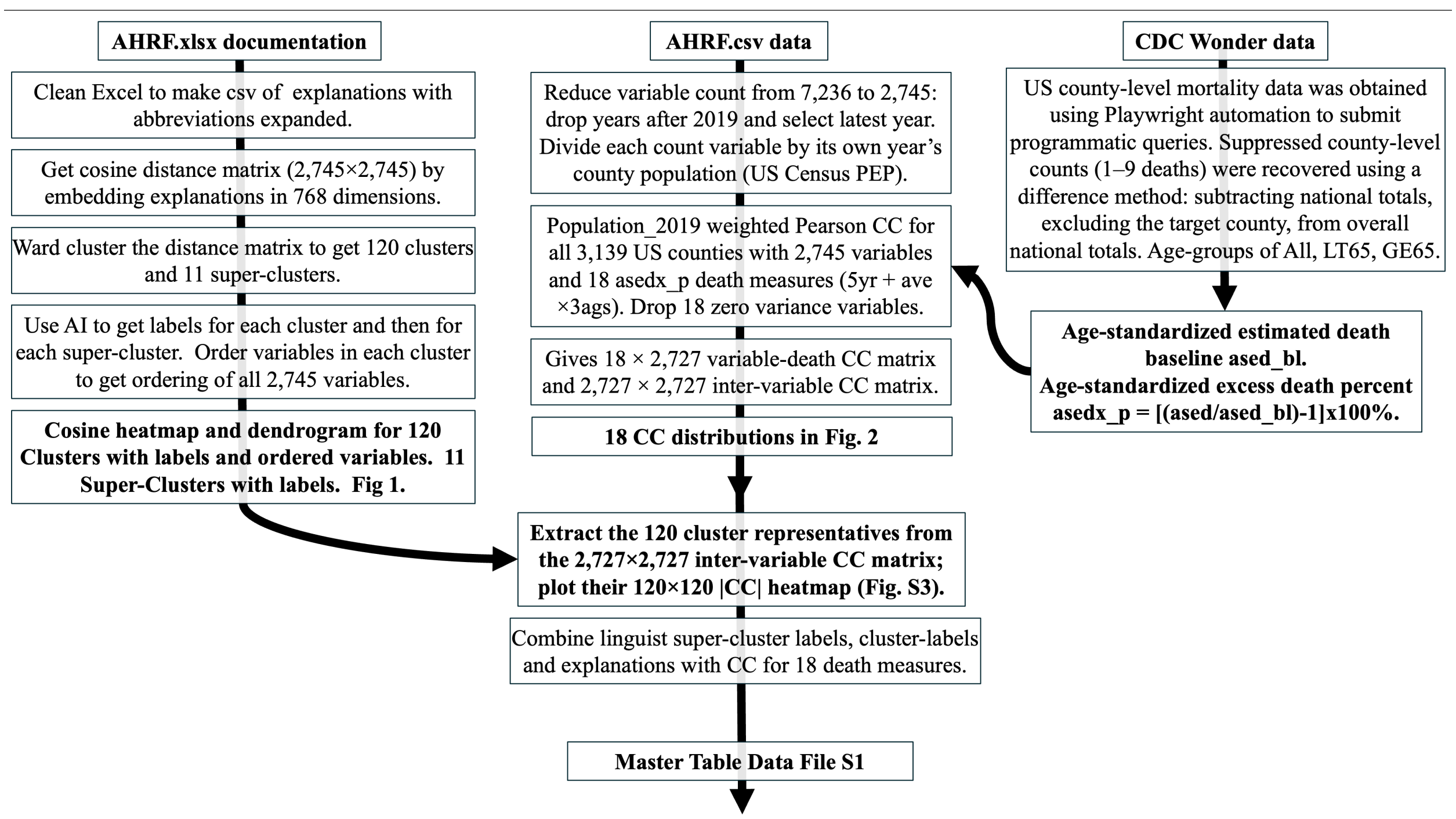

medRxiv preprint doi: https://doi.org/10.64898/2026.07.04.26357291; this version posted July 7, 2026. The copyright holder for this preprint (which was not certified by peer review) is the author/funder, who has granted medRxiv a license to display the preprint in perpetuity.
It is made available under a CC-BY 4.0 International license .

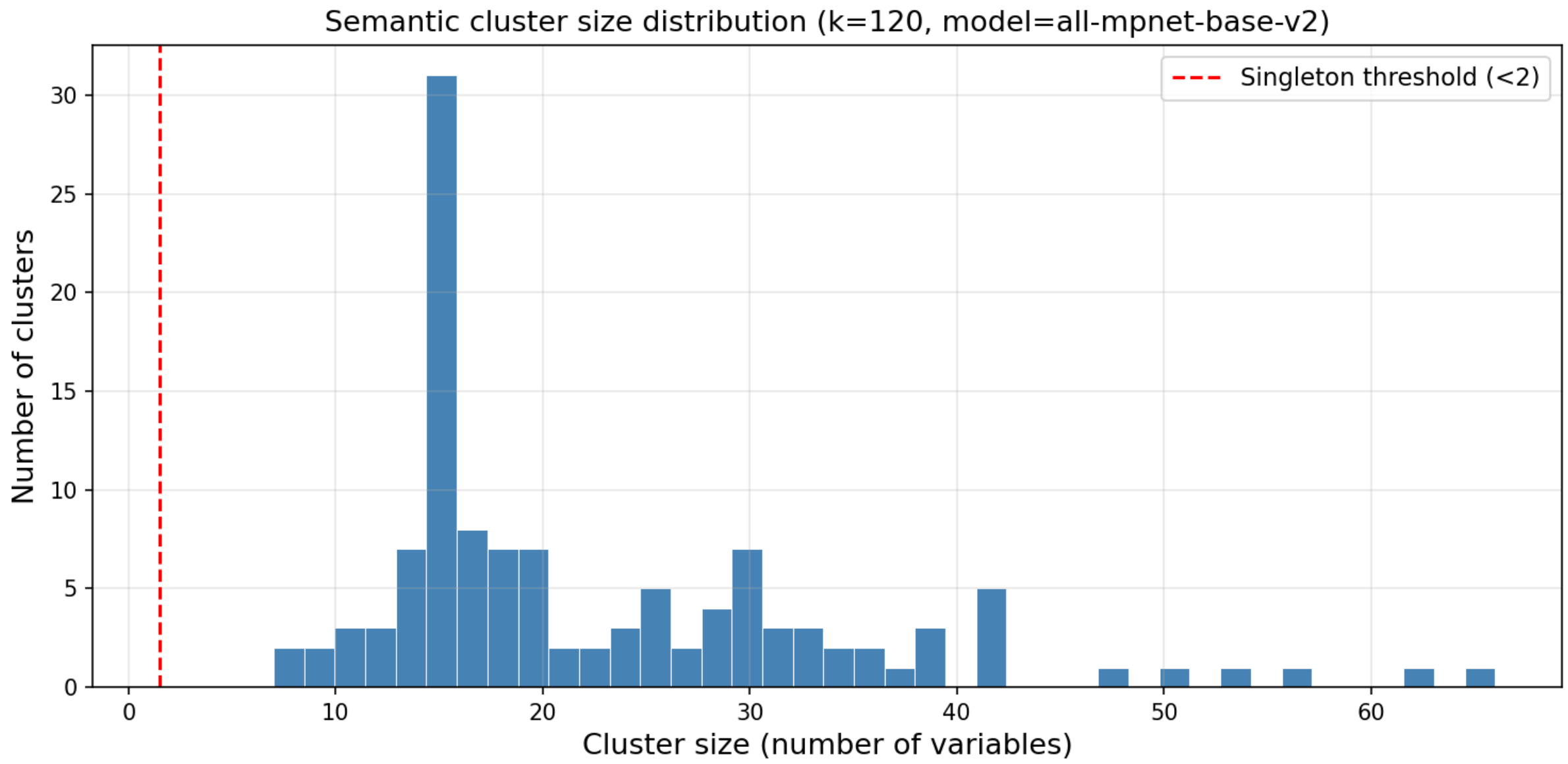


**Figure S2.** *Distribution of variable counts across the 120 semantic clusters (sizes 7–66 variables, median 18).*

medRxiv preprint doi: https://doi.org/10.64898/2026.07.04.26357291; this version posted July 7, 2026. The copyright holder for this preprint
(which was not certified by peer review) is the author/funder, who has granted medRxiv a license to display the preprint in perpetuity.
It is made available under a CC-BY 4.0 International license .

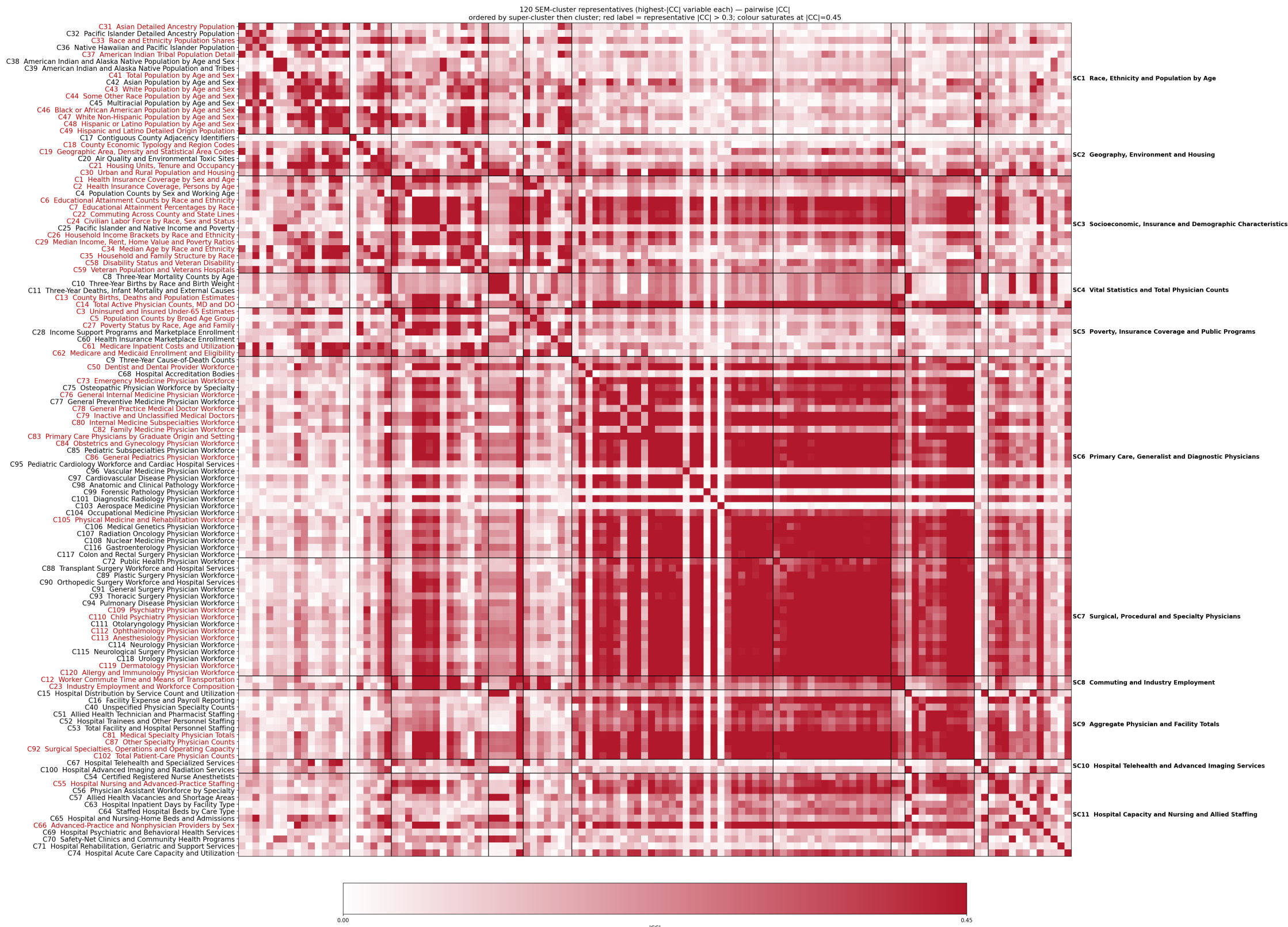


***Figure S3.*** *120×120 representative-variable |CC| heatmap (each cluster's highest-|CC| member); cell shading is |CC| magnitude (white→red, capped at 0.45). A cluster label is printed in red when its representative variable reaches |CC| > 0.30 (i.e. the cluster's strongest variable is moderately associated) — a looser criterion than Figure 1, which reddens a cluster only when ≥3 of its variables reach the moderate band in ≥2 years. The bright blocks are the demographic and socioeconomic super-clusters.*

medRxiv preprint doi: https://doi.org/10.64898/2026.07.04.26357291; this version posted July 7, 2026. The copyright holder for this preprint (which was not certified by peer review) is the author/funder, who has granted medRxiv a license to display the preprint in perpetuity.
It is made available under a CC-BY 4.0 International license .

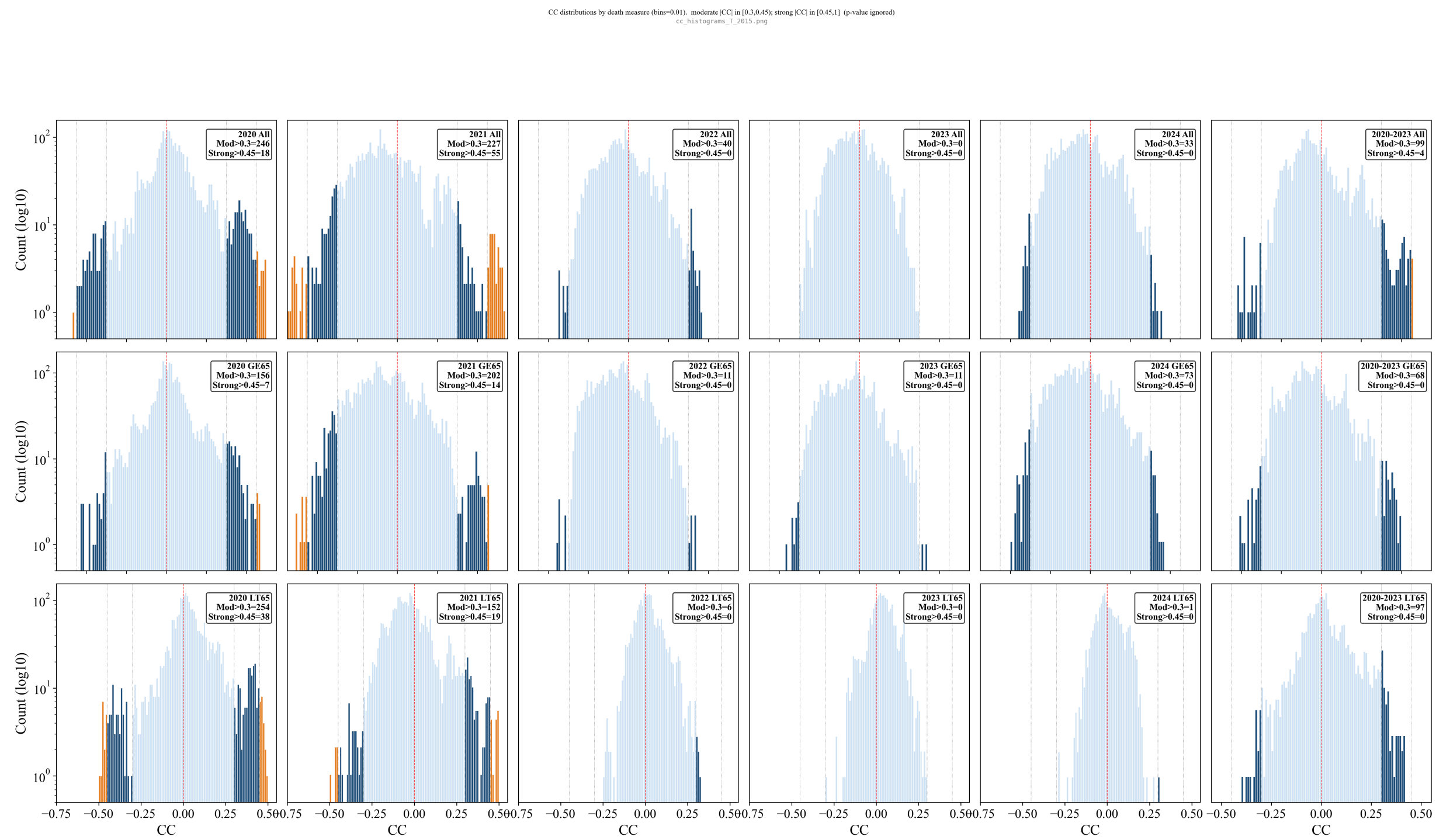


**Figure S4.** *Variable–death CC distributions for the 2015 backward-cutoff vintage (population-weighted). Columns: death years 2020–2024 plus pooled 2020–2023; rows: All / Ages ≥65 / Ages <65. $log_{10}$ count on the y-axis; dark blue = moderate (|CC| > 0.30), orange = strong (|CC| > 0.45); red line at CC = 0.*

medRxiv preprint doi: https://doi.org/10.64898/2026.07.04.26357291; this version posted July 7, 2026. The copyright holder for this preprint (which was not certified by peer review) is the author/funder, who has granted medRxiv a license to display the preprint in perpetuity.
It is made available under a CC-BY 4.0 International license .

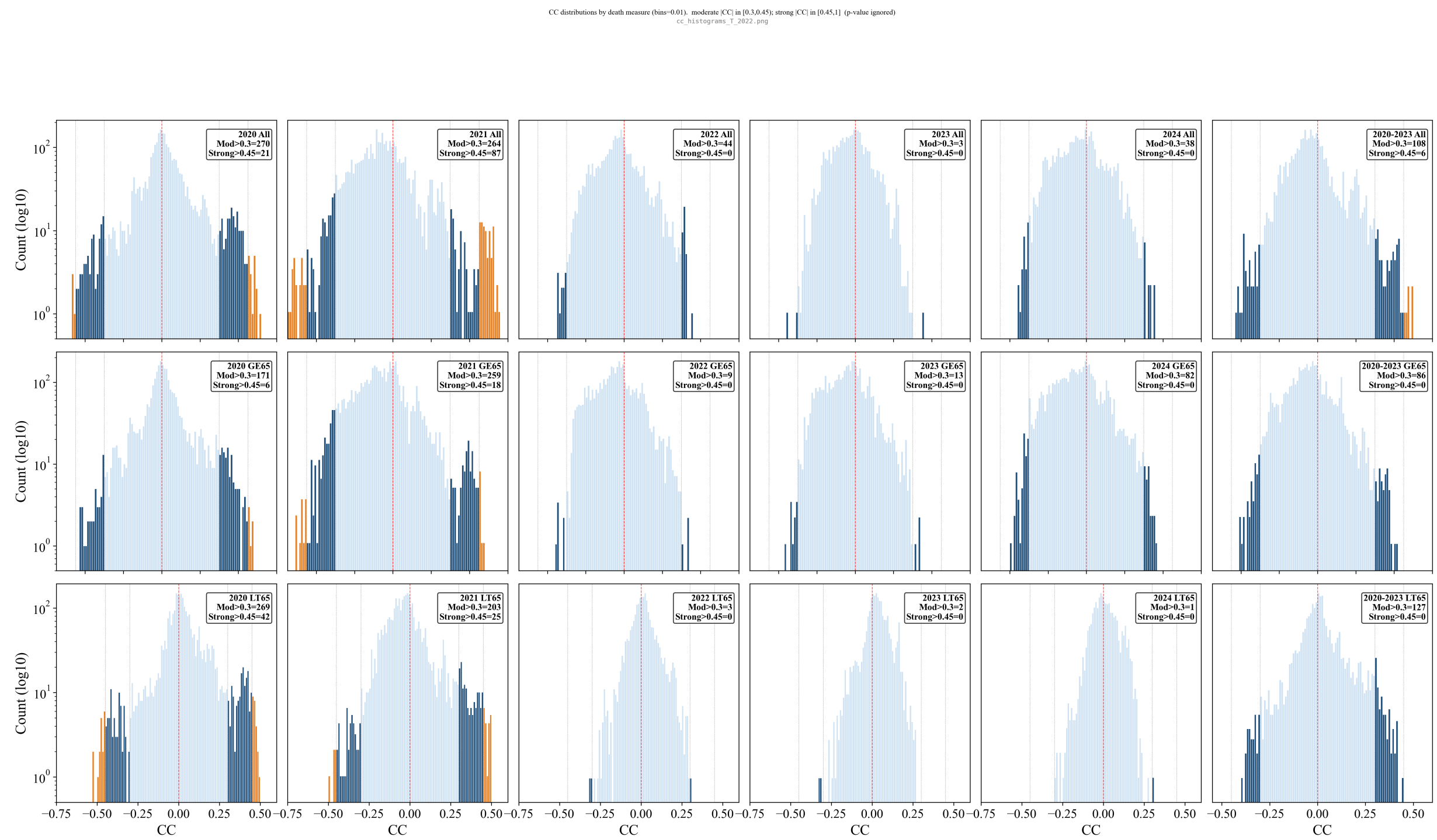


**Figure S5.** *Variable–death CC distributions for the AHRF 2023–2024 vintage, same axes and coloring as Figure S4. The moderate and strong tails are populated comparably to Figure S4 (2015) and to the 2019 standard run — the visual counterpart of the high variable-level vintage agreement (Table S2; Figure S6).*

medRxiv preprint doi: https://doi.org/10.64898/2026.07.04.26357291; this version posted July 7, 2026. The copyright holder for this preprint (which was not certified by peer review) is the author/funder, who has granted medRxiv a license to display the preprint in perpetuity.
It is made available under a CC-BY 4.0 International license .

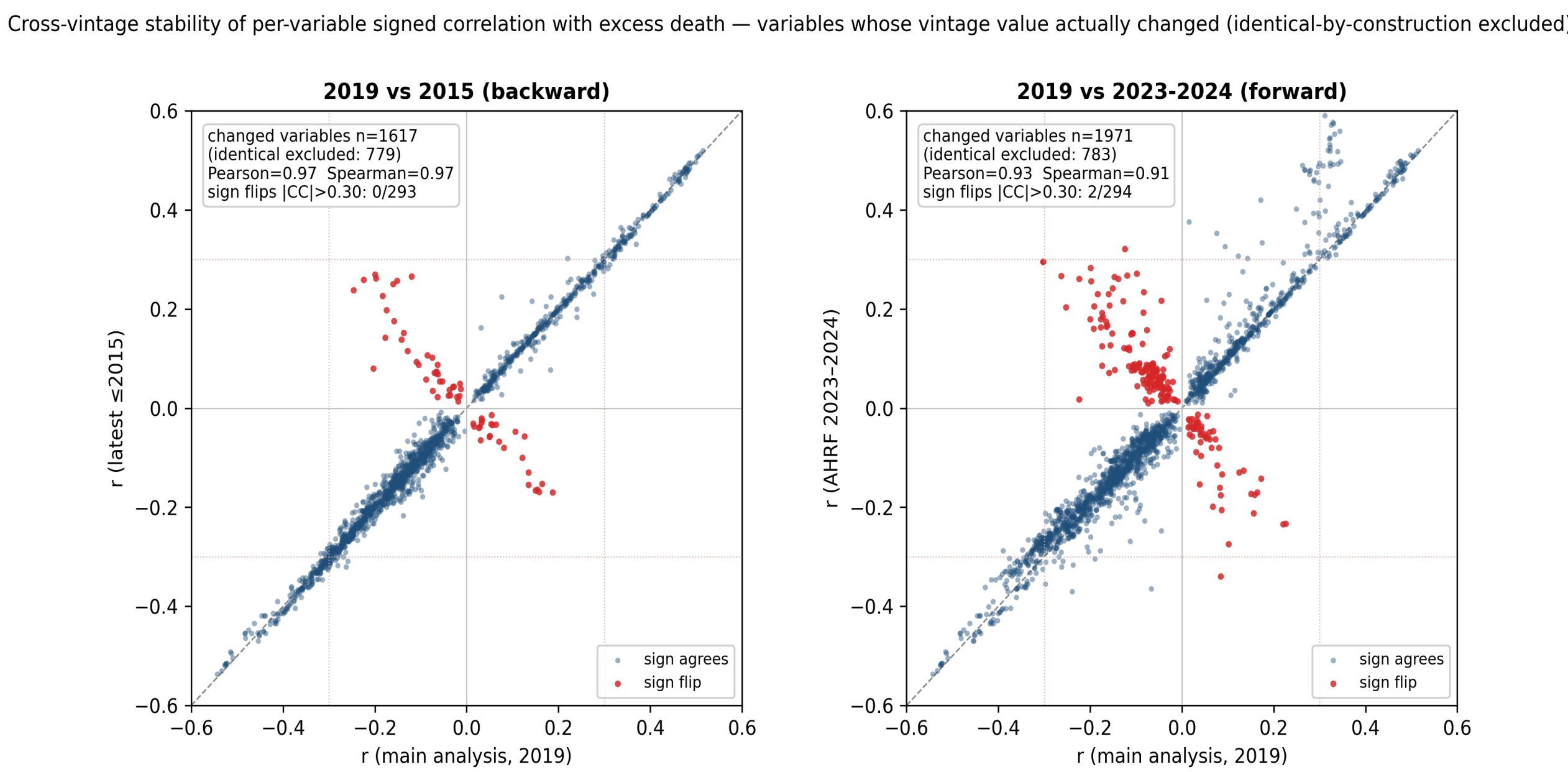


**Figure S6.** *Cross-vintage stability of the per-variable correlation with excess death. The comparison is restricted to the variables whose vintage value actually changed between releases: variables that are identical by construction — not rolled back (≤2015) or not refreshed (2023–2024) — are excluded as uninformative (1,068 excluded backward, leaving 1,328 tested; 1,137 excluded forward, leaving 1,617 tested). Each plotted point is one such variable; the axes are its signed correlation coefficient (the per-variable maximum |CC| over 2020–2024, with sign) in the main (2019–2020) analysis (x) versus the backward (latest year ≤ 2015; left panel) and forward (AHRF 2023–2024 release; right panel) sensitivity analyses (y). The dashed line is identity; dotted lines mark |CC| = 0.30. Points are red where the sign disagrees between vintages; among the variables reaching |CC| > 0.30 in the main analysis none reverse sign backward and only one does forward. Each panel annotates the Pearson and Spearman coefficients, the number of refreshed variables tested, and the number of identical-by-construction variables excluded.*
